# Mapping local structural perturbations in the native state of stefin B (cystatin B) under amyloid forming conditions

**DOI:** 10.3389/fnmol.2012.00094

**Published:** 2012-10-12

**Authors:** Robert Paramore, Gareth J. Morgan, Peter J. Davis, Carrie-anne Sharma, Andrea Hounslow, Ajda Taler-Verčič, Eva Žerovnik, Jonathan P. Waltho, Matthew J. Cliff, Rosemary A. Staniforth

**Affiliations:** ^1^Department of Molecular Biology and Biotechnology, University of SheffieldSheffield, UK; ^2^Department of Biochemistry and Molecular and Structural Biology, Institute Jožef StefanLjubljana, Slovenia; ^3^Faculty of Life Sciences and Manchester Interdisciplinary Biocentre, University of ManchesterManchester, UK

**Keywords:** Cu (II)-binding, precursors of amyloid, cystatin B, stefin B, proline isomerization

## Abstract

Unlike a number of amyloid-forming proteins, stefins, and in particular stefin B (cystatin B) form amyloids under conditions where the native state predominates. In order to trigger oligomerization processes, the stability of the protein needs to be compromised, favoring structural re-arrangement however, accelerating fibril formation is not a simple function of protein stability. We report here on how optimal conditions for amyloid formation lead to the destabilization of dimeric and tetrameric states of the protein in favor of the monomer. Small, highly localized structural changes can be mapped out that allow us to visualize directly areas of the protein which eventually become responsible for triggering amyloid formation. These regions of the protein overlap with the Cu (II)-binding sites which we identify here for the first time. We hypothesize that *in vivo* modulators of amyloid formation may act similarly to painstakingly optimized solvent conditions developed *in vitro*. We discuss these data in the light of current structural models of stefin B amyloid fibrils based on H-exchange data, where the detachment of the helical part and the extension of loops were observed.

## Introduction

The mechanism of amyloid fibril formation by human stefins A and B and their chimeras has been studied extensively by our groups (Kenig et al., 2006; Jelinska et al., 2011; Žerovnik et al., 2011). While stefin B amyloids have not been observed directly *in vivo*, mutations in the gene encoding stefin B cause a progressive form of Myoclonus Epilepsy (Unverricht-Lundborg disease or EPM 1) and it has been proposed that these mutations correlate with their aggregation behavior *in vitro* (reviewed in Polajnar et al., 2012). Compared with its better recognized partner cystatin C, which causes a hereditary cerebral amyloidosis, it is more readily available for biophysical work and, as such, has been extensively used as a working model. While structural studies have focused on the amyloid endpoint (Morgan et al., 2008), kinetic studies have led to a proposed model for the amyloidogenesis of stefin B (Skerget et al., 2009). This protein was shown to conform to other globular amyloid forming proteins in both the morphology of final amyloid fibrils and in the early appearance of the prefibrillar oligomers which range from dimers, tetramers, and hexamers to higher oligomers—such as 8-mers, 12-mers, 16-mers to even 32-mers (Ceru et al., 2008). These oligomers have been extensively studied and proved to behave as other “amyloid toxins,” interacting with lipid membranes and even making pores (Ceru et al., 2008; Rabzelj et al., 2008).

Knowledge of the aggregation behavior of proteins, particularly in the context of the formation of fibrous amyloid-like structures, has widened our original simplistic unimolecular view of the energy landscape for a protein (Dobson, 2003; Eichner and Radford, 2011). Although the original hypothesis proposed by Levinthal (1969), that a protein folding pathway must exist, is still valid, the complexity of this pathway and our ability to define it have been challenged. Amyloid is an aggregate that invariably requires a substantial structural re-arrangement within the naturally occurring protein precursor (Jarrett and Lansbury, 1993; Wetzel, 1996; Rochet and Lansbury, 2000). It is pertinent to the current study, that we should examine the mechanism through which proteins from the cystatin family, and in our example, stefin B (also referred to as cystatin B) is channeled down alternative folding routes.

Current thinking supports the idea that the folding of small regions may be significantly important in fibril formation. In protein folding the nucleation condensation model (Fersht, 1995) suggests that only a small number of contacts may be required to initiate protein folding. In the protein aggregation field, the idea of amyloidogenic determinants (the minimal regions required for fibril formation) predominates and the arrangement of very short sequences is believed to drive the formation of fibrils (Conchillo-Sole et al., 2007; Tartaglia et al., 2008; Maurer-Stroh et al., 2010). Local disturbances that are independent of or uncoupled to the folding of the protein as a whole may lead to alternative folded states. This type of non-cooperative behavior has been observed before in the folding of “uncoupled proteins,” where removal of a core interaction causes folding in small regions at a time [e.g., apomyoglobin (Staniforth et al., 2000) and Ca^2+^-free α-lactalbumin (Schulman et al., 1997)].

The alternative hypothesis is that simply destabilizing the native fold by shifting the equilibrium of unfolding would be sufficient to induce fibrillization. This hypothesis is not supported when calculations of fibrillization propensity are conducted (Chiti et al., 2003). These calculations necessarily include many parameters with the ability to form β-sheet structure being the most influential. Also mutational studies on stefin B show that the stability of the protein does not correlate with its fibrillization propensity (Kenig et al., 2006).

In this work, we use NMR spectroscopy to examine structural changes in soluble forms of stefin B. Under the conditions of biochemical experiments, where Stefin B concentrations are at least 1 μM, the protein naturally populates different oligomeric states and the recombinant protein is obtained as a mixture of monomers, dimers, and tetramers from *E. coli*. We report here on the multistate nature of the dimeric form of the protein, which is believed to be the precursor to amyloid. We then map out regions of the protein which are perturbed when the protein is incubated under the amyloidogenic conditions which have been optimized *in vitro* (Zerovnik et al., 2002). Finally we compare these artificial conditions to the effects of the divalent metal Cu (II) which is known to interact with stefin B (Zerovnik et al., 2006).

## Materials and methods

### Protein and amyloid fibril production

Our Stefin B construct (C3S, with glutamate polymorph at position 31) was transformed into *E. coli* BL21-DE3 cells and was purified according to Rabzelj et al. (2005) and Morgan et al. (2008). Stefin B amyloid fibrils were grown from 30 μM soluble protein by incubating at 30°C in 15 mM sodium acetate buffer, pH 4.7, containing 150 mM NaCl and 10% trifluoroethanol (TFE). CuSO_4_ was added according to the ratios indicated to a final concentration of 15 μM, 30 μM, and 60 μM. Fibril growth over time was monitored by adding thioflavine T to a final concentration of 10 μM (Naiki and Gejyo, 1999). Fluorescence spectra (λ_ex_ = 442 nm and λ_em_ = 482 nm) were recorded on a Shimadzu RF-5301PC (Shimadzu, Japan) or a Varian Cary Eclipse (Agilent, UK). The fluorescence amplitudes of the reactions were normalized as the presence of Cu (II) affects the total fluorescence value. Because the formation of amyloid fibrils by stefin B is largely independent of protein concentration (Skerget et al., 2009), time courses were fitted to a single exponential increase to determine the elongation rate of the fibrils. Lag times were deduced from the cross-over point with the x-axis. All data manipulation and fitting was carried out using GraFit, Version 3.0 (Erithacus Software Ltd.).

### Size-exclusion chromatography (SEC)

The oligomeric state of protein samples was analyzed using a KW-803 size exclusion column (Shodex, Japan) attached to a Perkin Elmer Series 200 HPLC system with a UV absorbance detector. The buffer used was 50 mM sodium phosphate buffer, 100 mM NaCl, pH 7, 1mM NaN_3_ for native samples and while pH titrations were carried out in 15 mM sodium acetate, 150 mM NaCl, 1mM NaN_3_ at a range of pHs between 6.0 and 4.7.

### NMR spectra

Samples for NMR in native conditions were made by adding ^2^H_2_O [final concentration 10% (v/v)] to a 500 μl sample with a uniformly labeled ^15^N protein concentration of 200 μM (monomer equivalents), as determined by UV absorption, in 10 mM sodium phosphate buffer (pH 6) and containing 100 mM NaCl (the conditions in which it was purified by gel-filtration chromatography). pH titrations were carried out in 15 mM sodium acetate buffer 100 mM NaCl at a range of pHs between 6.0 and 4.7. All spectra were recorded at 25°C on Bruker DRX spectrometers operating at proton frequencies of 500 MHz, 600 MHz, or 800 MHz, using either room temperature or cryogenically cooled triple-resonance probes. A combination of in-house and standard Bruker pulse programs was used. NMR data were processed using Felix 2000 and Felix 2004 software (Accelrys, CA, USA) running in-house macros.

### Assignment of the cystatin B dimer ^1^H^15^N HSQC spectrum

Samples were made up as above but protein concentrations were 1 mM and both ^15^N and ^13^C labeling of the protein was required. NMR backbone resonance assignment was carried out as previously described (Morgan et al., 2008). Where extra peaks complicated the assignment procedure in which we used the “Asstools” software (in-house program of the Leicester University Biological NMR centre), it was necessary to determine which peaks were equivalent—representing the same residue—by comparing their carbon chemical shifts. Peaks near to each other which had very similar sets of carbon cross-peaks were likely to represent the same amide, especially if their positions were similar to the assigned peak from the ^1^H^15^N HSQC spectrum of the monomer. The duplicate peaks were excluded from the spin system list. This strategy allowed the peak list to be reduced to a more manageable number, and most of the residues have been assigned. The exceptions are glycine 60, glutamate 62, and aspartate 63, although aspartate 61 is still identifiable. That these peaks are missing is consistent with the exchange broadening associated with the conformation of glycine 60, lysine 33, and aspartate 63 described.

### pH and TFE titrations

Proton and nitrogen chemical shifts for each residue were extracted from ^1^H^15^N HSQC spectra and peak position change was calculated with respect to peak position at pH 6.0, 0% TFE. A threshold for significance was set to be two times the average line width (width of the peak in ppm) at 50% peak height. This value is 0.075 ppm for proton and 0.56 ppm for nitrogen chemical shifts. Data for the movement of peaks in the dimeric form show the average of the absolute movement for each residue as most residues display at least two peaks in the ^1^H^15^N HSQC spectra. So that movements in opposite directions do not cancel each other out the absolute values for movement were used during data analysis and the movement of multiple peaks was averaged to a single value.

### Cu (II) titrations

The peak heights for assigned residues were extracted from 2D ^1^H^15^N-HSQC spectra of a Cu (II) titration experiment for both the monomeric and dimeric states of stefin B. Since there is an overall reduction in peak height it is necessary to decide what is classed as a larger than normal loss in peak height. To decide upon this, the standard deviation of the mean peak height loss of all residues was calculated and all data greater than one standard deviation of the mean were taken as significant.

### Circular dichroism

Equilibrium unfolding of stefin B was monitored at a range of CuSO_4_ concentrations (ratios of 0:1, 1:1, and 5:1 Cu (II): protein). 50 μM protein solutions were prepared at a range of guanidine hydrochloride (GdnHCl) concentrations in fibrillization buffer minus the TFE (15mM sodium acetate pH4.7, 150mM NaCl). Circular Dichroism spectra were recorded at 25°C on a Jasco 810 spectropolarimeter (Jasco, Japan) and the titration data fitted according to Jelinska et al. (2011).

## Results

### Stefin B in solution is a mixture of monomer, dimer and tetramer

As shown before (Ceru et al., 2008) recombinant stefin B is purified from *E. coli* as a mixture of monomer, dimer, and tetramer. The monomeric state is only meta-stable under most experimental conditions (ionic strength > 100 mM, 6.5 < pH < 8.0) and will readily form dimers within minutes (Zerovnik et al., 1997; Kenig et al., 2006; Jelinska et al., 2011). Because of the unusual 3D domain-swapped nature of the dimers, it is unlikely that the dimers can revert back to monomers without first undergoing an unfolding reaction (Scheme I, Jerala and Zerovnik, 1999; Staniforth et al., 2001). Tetrameric forms of the wild-type stefin B protein can be purified but re-equilibrate over a time-scale of 15 h at room temperature to a mixture in which they populate only a minority fraction (Jenko Kokalj et al., 2007) even at artificially high protein concentrations (>4 mM). Under a chosen set of conditions, the population of any particular oligomeric state of stefin B is therefore a competition between the rates of unfolding, dimerization, and folding. This has been quantified for another more amenable cystatin from chicken egg white (Sanders et al., 2004).

Previous work reported that the NMR spectra of the folded states of stefin B are very similar, indicating that the basic fold of the protein is not significantly changed between the monomer, dimer, and tetramer (Jenko Kokalj et al., 2007; Morgan et al., 2008). The changes observed between monomeric and dimeric stefin B map onto the regions of the structure involved in dimerization by 3D-domain swapping, with the largest changes around the loop between strands 2 and 3, which forms the hinge loop in the domain-swapped structure of stefin A. Other residues whose chemical shifts change substantially form the interface between the two domains of the dimer and include the loop between strands 4 and 5 (for e.g., Figure 4B). This is reminiscent of changes observed for its homolog, stefin A, upon dimerization (Jerala and Zerovnik, 1999; Staniforth et al., 2001). Chemical shift differences between the dimer and tetramer of stefin B occur in the same regions as those observed between the monomer and dimer where further structural changes lead to tetramer formation (Jenko Kokalj et al., 2007).

In this study, we were interested in mapping structural changes observed when the protein is induced to fibrillize into amyloid. We therefore examined the oligomeric state of the protein under our experimentally optimized conditions (Zerovnik et al., 2002) which are mildly acidic (pH 4.7) and contain 10% TFE, a solvent which is known to aid fibrillization (Chiti et al., 1999), presumably because of its dual nature as both a denaturant and a secondary structure stabilizing agent. These conditions were those used in our previous structural characterization of stefin B amyloid and are pertinent here.

**Figure 1:**
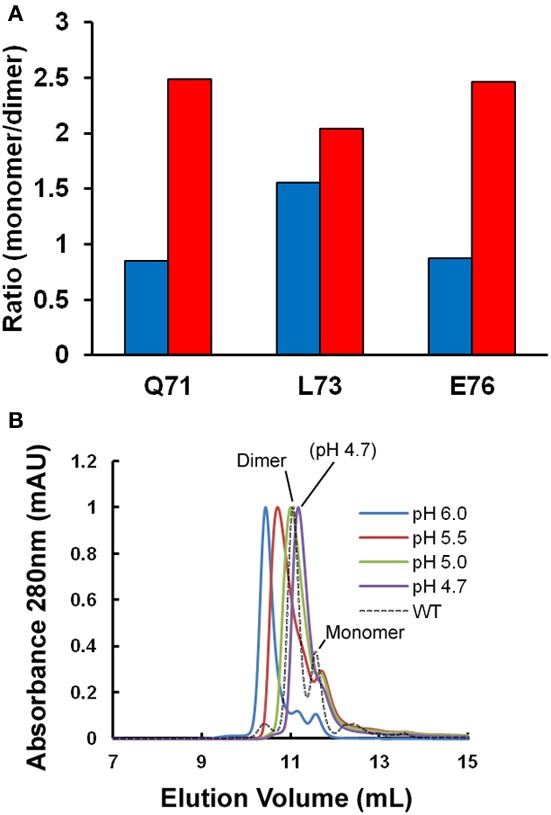
**Oligomeric state of stefin B under fibrillization conditions *in vitro*. (A)** Changes in the apparent ratio of monomer to dimer as the protein is taken from physiological pH (pH 6.0, in blue) to conditions which favor fibrillization *in vitro* (pH 4.7, in red). The ratio of monomer and dimer is calculated from amide peak intensities for three residues (Q71, L73, and E76) showing well-resolved NMR signal changes in the 2D ^1^H^15^N HSQC. Under the conditions of the experiment, we estimate the error to be ±0.25. **(B)** A single point mutation (P79S) causes the stabilization of the tetrameric state of stefin B in solution at pH 7.0 (Jenko Kokalj et al., 2007). Fibrillization conditions optimized *in vitro* cause dissociation of the tetramer to a mixture of dimer and monomer close to WT, which explains how this mutant remains a slow fibrillizing species.

Figure 1A shows the ratio of monomeric to dimeric stefin B under the near-native conditions in which it is purified (in blue) and as the pH is lowered to pH 4.7 and TFE titrated to 10% (in red). Although the error on the data is high (±0.25), it is of note that the ratio of monomers to dimers is observed consistently to increase from a 1:1 to a 2:1 ratio. This is because although the dimeric form of the protein may be energetically more stable than the monomer; the two species never reach equilibrium. In order to understand why this is, it is useful to consider that the system is composed of more than just two states (Scheme I). Under fibrillization conditions, the population of unfolded states (U) has been shown previously to increase greatly and can comprise up to 1% of the sample (Zerovnik et al., 1998, 1999, 2007; Jelinska et al., 2011). Increasing the unfolding rate of dimers (red arrow) will allow a greater population of folded monomeric states to accumulate at any point in time:

**Figure:**
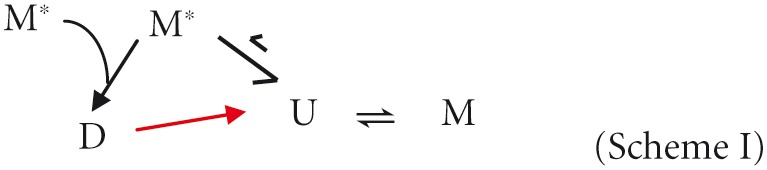


As the dimer D unfolds to form U, it dissociates and then always forms the monomer M more rapidly than it can dimer. This is simply because any part of a protein chain is more likely to find its intramolecular partner residues before those in a different molecule unless the protein concentration in solution exceeds ~10 mM (estimate of effective concentration according to Creighton, 1993). As was proposed for its chicken cystatin counterpart (Sanders et al., 2004), and based on current data for our stefin B protein, we can hypothesize that the formation of the dimer requires the coming together of two, relatively rare, partially folded states of the protein (M^*^) in an orientation that is “assembly-competent.”

The tetrameric state of the wild-type protein is only a minor component at the temperatures greater than 20°C used here for NMR so we exploited the effect of a mutation at proline 79 to serine (P79S) in order to examine the effect of fibrillization conditions on this oligomeric form of stefin B. This mutation favors the tetrameric form of the protein and in fact allowed crystallization and structural determination of this state previously (Jenko Kokalj et al., 2007). Figure 1B shows the effect of lowering the pH to 4.7 on this oligomeric form of the protein as measured using size-exclusion chromatography. The elution peak shifts suggesting there is structural exchange on the timescale of the experiment. Importantly, at pH 4.7, the P79S stefin B variant shows a profile where there is no detectable tetrameric stefin B in solution and the dimeric form predominates. The tetrameric form of stefin B has been suggested as a precursor to amyloid formation but under the fibrillization conditions used *in vitro* at least, this state is not populated significantly. This is also the case for other variants of this protein where the tetrameric forms remain undetectable under fibrillization conditions, unlike its homolog from chicken egg white (Sanders et al., 2004 and manuscript in preparation).

To conclude, the principle forms of stefin B populated under our *in vitro* fibrillization conditions (pH 4.7 and 10% TFE) are monomers and dimers. We will therefore focus on these molecular species of stefin B to examine early structural changes leading to fibril formation.

### Multiple states in the dimer

Proteins in solution do not have a fixed, rigid structure, but are flexible, dynamic molecules, which can adopt many different conformations. The NMR signal or chemical shift of a particular nucleus depends on the conformation of the protein. If the protein has a single structure which it occupies most of the time, then the observed chemical shift will correspond to that structure. If, however, the nucleus sees more than one environment, corresponding to different protein conformations, then the different chemical shifts will contribute to the spectrum. The major influences on amide chemical shifts in proteins are residue conformation, hydrogen bonding and nearby aromatic groups. The dispersion of proton chemical shifts in a 2D ^1^H^15^N-HSQC NMR spectrum is mainly due to hydrogen bonding patterns defined by secondary structure.

**Figure 2:**
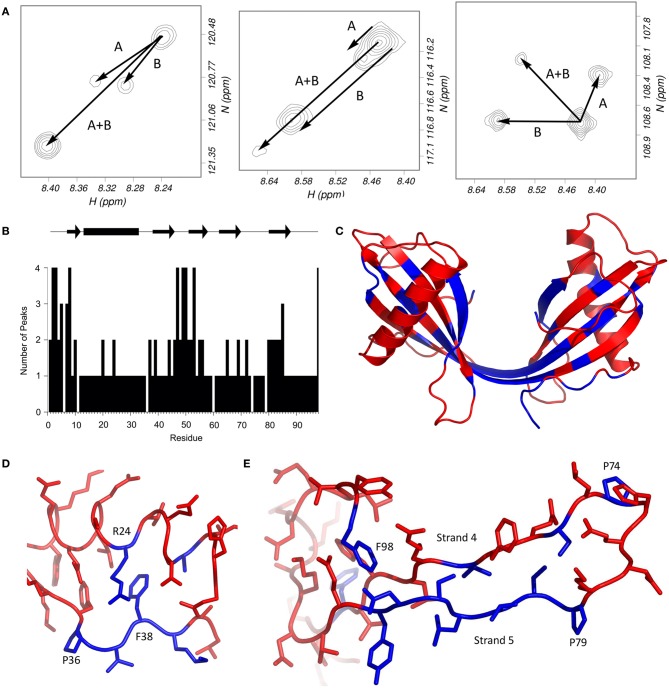
**Multiple HSQC peaks in the spectrum of the stefin B dimer. (A)** Pattern of multiple peaks in the HSQC spectrum. To illustrate, the four peaks assigned to each of methionine 2 (left), serine 7 (centre) and glycine 50 (right) suggest that two conformational changes (labeled A and B) can account for the four states. **(B)** The number of peaks in the HSQC are plotted against residue number. **(C)** Residues with multiple peaks are colored blue in the structure of the dimer (bottom). **(D)** Multiple HSQC peaks around proline 36. In this view of the stefin B monomer (PDB code 1STF), residues with multiple peaks (and proline 36) are blue; other peaks are red. Proline 36 is in the *trans* conformation, and isomerization could affect the conformation of residues 37–39, as well as arginine 24, which packs against phenylalanine 38. The resulting two structures could give rise to the two peaks seen for each blue residue in the HSQC spectrum of the dimer. **(E)** Multiple HSQC peaks around strands 4 and 5. The loop between the two C-terminal β-strands of stefin B is part of the protease binding site, and is flanked by two proline residues at positions 74 and 79. *Cis*-*trans* isomerism in proline 79 could be the cause of the multiple peaks observed on strand 5, which could in turn affect phenylalanine 98. The two prolines, and residues which have multiple peaks in the HSQC spectrum of the dimer are blue; other residues are red.

The 2D ^1^H^15^N-HSQC spectrum of the dimer of stefin B shows unusual features which are not shared with other oligomeric forms of the protein. A large proportion of the residues in the protein have more than one peak attributable to them (Figure 2A). How a residue's conformations influence its NMR spectra depends on the rate at which the protein changes its conformation. Most local fluctuations within proteins occur too quickly to observe directly by looking at different chemical shifts. However, if the change in conformation is slow compared to the chemical shift difference (generally less than 10 per second, a halftime of 100 ms), then the conformations can produce discrete peaks, if the difference in chemical shifts is large enough to be resolved. The discrete peaks seen in the spectrum of the dimer (Figure 2A) suggest that the protein populates at least four distinct conformations, and the exchange rates between these states are slow, indicating large energy barriers between them. An alternative explanation is that the protein forms an asymmetric (and therefore probably not 3D domain-swapped) dimer. However, only a third of the protein's residues have more than one peak, which suggests that much of the structure is the same for the two domains (Figure 2B).

Looking at the residues which have several peaks (Figure 2A), it is possible to observe patterns in the distribution of the peaks. Generally, there seem to be four peaks for each of the “split” spin systems, which generally have the same carbon shifts, suggesting that the structure at these positions is similar. Glycine 50 is a good example of the pattern of peaks; it seems to be a doublet of doublets, suggesting that there are two distinct changes causing the four populations. The pattern is repeated for several other residues, including S3, S7, and F98. The existence of small but significant energy barriers between the different species and this splitting behavior is reminiscent of the behavior of residues located near proline residues where flexible structure allows cis-trans isomerization processes to occur.

### Lowering the pH

In 2D ^1^H^15^N-HSQC experiments, peak positions (chemical shifts) are highly sensitive to changes in the chemical environment of the corresponding residue. We used this experiment to detect even small changes in the structure of monomers and dimers as fibrillization conditions are titrated in. To start with, we carried out a pH titration. This was done in small pH steps (~0.3–0.4 pH units) to allow peak movements to be tracked with confidence, but even then, some peaks moved beyond the point that they could be followed. N77 and H75 became indefinable for the monomeric and dimeric structures respectively between pH 5.0 and pH 4.7. Large movements like this are generally due to changes in protonation state as a result of moving though the intrinsic pK_a_ of particular residues.

**Figure 3:**
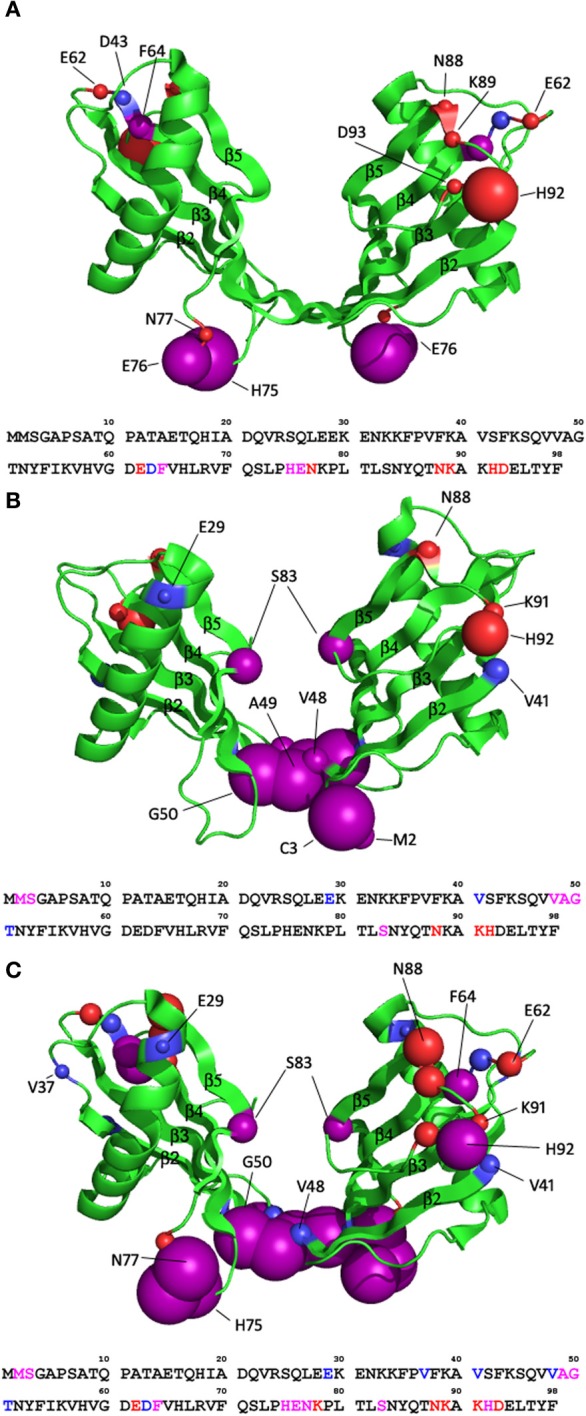
**How *in vitro* fibrillisation conditions affect the structure of the stefin B dimer (pdb code 2OCT). (A)** The effect of lowering the pH from 6.0 to 4.7. **(B)** The effect of titrating in 10% TFE at pH6.0. **(C)** The effect of combining these two effects and transferring the protein to fibrillisation conditions, pH4.7, 10% TFE. The residues of stefin B that are significantly affected are shown as spheres with a radius proportional to the change in chemical shift observed (0.075–0.2 ppm in proton shift or 0.55–1 ppm nitrogen shift). Purple spheres represent residues with significant change in both proton and nitrogen chemical shifts while red and blue represent residues with significant change in proton or nitrogen only chemical shifts respectively.

Whereas six residues display significant movement in either proton or nitrogen chemical shift in the spectrum of the monomeric stefin B, for the dimeric structure, a larger number of residues are seemingly affected by the pH titration compared to the monomeric state. Four residues show significant nitrogen chemical shift change while nine show significant proton chemical shift change. Of the five additional residues highlighted in the dimer compared to the monomer, two (62 and 63) do not have assignments in the monomeric form (so no data is available on their movement) while two (64 and 93) show large proton chemical shift changes (−0.074 and 0.071 respectively) that lie just under the threshold of 0.075 set for this data. For these reasons, only the dimer structure is shown in Figure 3A to illustrate these effects.

There are two regions within the monomeric form showing significant change during the pH titration from pH 6.0 to pH 4.7. The change in residues L73, E76, and N77 can be explained by the protonation of residue E76. The locations of E76 and L73 in the monomer suggest the possibility of a hydrogen bond between them, but due to flexibility in this region the crystal structure cannot give the exact positions of these residues. Protonation of E76 would prevent the hydrogen bond from forming. Without the constraining hydrogen bond, E76 could move out into solution, changing the dihedral angle of the peptide backbone, which in turn will alter the chemical shift of N77. Also, the protonation of E76 would affect the nitrogen chemical shift of N77, which is reflected in the data. The proton chemical shift of N77 will be affected by N-H bond rearrangement.

The change in proton chemical shift of the other region (88–93) will be the result of N-H dihedral bond angle change and not protonation since the nitrogen chemical shifts are not affected. There is already flexibility in the loop regions (reflected by quite large b-factors in the crystal structure (PDB 1STF, Stubbs et al., 1990) so slight rearrangements will not alter the overall fold of the protein.

In the dimeric structure, the region around E76 is again affected by chemical shift change in nitrogen and proton dimensions, as a result of protonation of the glutamic acid at position 76. L73 is not in the correct orientation in the dimer to form a hydrogen bond with E76 so is not affected. There is an increase in the number of affected residues in the 88–93 region in the dimer compared to the monomer. This will be in response to changes in the geometry of the N-H bonds in this region because only proton chemical shifts are affected.

Residues 62–64 show significant chemical shift change in the dimer but not in the monomer, this is because of the lack of an assignment for these residues in the monomer rather than a different behavior occurring in the monomer. Residues 62–64 are displaying chemical shift change because of the glutamic and aspartic acid residues in the region. The most likely candidate for protonation is residue D63 since this and the following residue (F64) both display a change in nitrogen chemical shift. This may result in them becoming more solvent exposed, thus in turn altering backbone dihedral angles explaining the H chemical shift change in E62 and F64.

Overall the areas in the monomer and dimer affected by dropping the pH from 6.0 to 4.7 are the same. The chemical shift changes observed are restricted to the mobile loop regions between strands 3 and 4, 4 and 5 and the C-terminus. There is thus no evidence for large scale (global) structural changes associated with a partially folded intermediate state.

### Effect of trifluoroethanol (TFE) on the native stefin B structure

Previously, circular dichroism data (Zerovnik et al., 1999) suggested that concentrations of TFE lower than 15% v/v have little or no effect on the folded state of the protein. The concentration of TFE in the fibrillization buffer used for stefin B is 10% v/v. Without the addition of TFE, the fibrillization process takes months instead of weeks to occur (Zerovnik et al., 2002).

To investigate the possibility of minor structural alterations being propagated by the addition of TFE, 2D ^1^H^15^N-HSQC spectra were recorded for a titration from 0 to 10% TFE at pH 6.0. TFE has very little effect on the chemical shifts of the monomer. Four residues show significant change in the nitrogen dimension (E29, V41, V48, and S83) but no residues show significant change in proton chemical shift. When these residues are plotted onto the structure they are not confined to a specific region or structural element, nor are they amino acids of similar chemistry suggesting these effects are unlikely to be truly significant. Inspection of data collected on the dimer shows TFE has a more significant effect on the dimeric state of stefin B. Six residues show chemical shift change in both dimensions, three show nitrogen chemical shift change only and three show proton change only (Figure 3B). There is an obvious cluster around the extended β-sheet between the two domains of the domain swapped dimer, comprising strands 2 and 3 of the original monomer fold. This region shows chemical shift change in both proton and nitrogen dimensions suggesting large structural changes in this region may be occurring. This region has great flexibility which could easily cause significant chemical shift change. Other than this region, residues showing change are scattered around the rest of the structure and are probably the result of localized structural changes in loop regions. Other than the flexibility in the extended β-strand region (comprising strands 2 and 3), there is very little chemical shift change, suggesting that there is no structural change occurring to the general fold of the dimer.

Overall, it can be concluded that the change in chemical shift in the extended β-strand region is the result of destabilization of that region, which in turn causes a destabilization of the dimeric form (Figure 1A). This area is the “hinge” region which bridges the two cystatin fold units in the 3D domain-swapped dimers of cystatin B. Destabilized dimers most likely dissociate then refold back to a monomeric form which is less affected by the presence of TFE (see Scheme I in earlier section). This provides a structural explanation for the observation of dimer dissociation upon addition of TFE (section “Stefin B in solution is a mixture of monomer, dimer and tetramer”).

### Effect of the combination of reducing pH and the addition of TFE on native stefin B

The effect of reducing the pH and adding TFE on the structure of stefin B has been assessed on an individual basis in the previous two sections. However, the conditions required for stefin B fibrillization call for a combination of decreased pH and addition of TFE. To check that this combination does not alter the structure of stefin B to a larger degree than the conditions individually, the chemical shifts for the monomer and dimer were extracted from an ^1^H^15^N HSQC spectrum of stefin B in 15 mM sodium acetate pH 4.7, 10% v/v TFE. The chemical shifts from this spectrum were compared to the chemical shifts at pH 6.0, 0% v/v TFE. The monomeric form has a total of 15 residues exhibiting a significant chemical shift change while the dimeric form has 22. Plotting these residues onto the structures of the stefin B monomer and dimer shows dispersion of the residues across the structure of the monomeric form, although they could be said to be loosely confined to the loop regions at the ends of the β-strands. The dimeric form shows the same pattern and is thus shown for illustration in Figure 3C. Extensive chemical shift change is localized to the ends of loops, but with the addition of the extended β-sheet region between the two domains of the dimer (strands 2 and 3).

### Mapping the effect of Cu (II)

Previous work showed that stefin B monomer and dimer, both of the wild type (WT) E31 and a Y31 variant, bind Cu (II) ions (Zerovnik et al., 2006), whereas the tetramer (P79S) did not bind this ion. Divalent metal ions such as Cu (II), Zn (II), and Fe (II) are observed to be co-localized in amyloid plaques *in vivo*. The concentration of these metals is often much higher than normally found within the human body, ~400 μM, ~1 mM, and ~1 mM for Cu (II), Zn (II), and Fe (II) respectively (Curtain et al., 2001). This massive increase in localized concentration has led to the hypothesis that these metals are bound by the mature amyloid fibrils and that they may influence the formation of amyloid fibrils.

NMR was used to perform 2D ^1^H^15^N-HSQC experiments to identify regions within the native stefin B oligomers (monomer and dimer) that bind, or are close to the binding sites of Cu (II). Cu (II) has a very definite effect when looking at protein by NMR due to its paramagnetic nature. When a protein binds Cu (II), the lone electrons interfere with the magnetic dipoles in close vicinity. This shields them from the magnetic field generated by the NMR magnet and so causes them to become undetectable by solution NMR. We therefore identified the specific residues that are close to the Cu (II) binding site by monitoring their disappearance in a series of 2D ^1^H^15^N-HSQC spectra collected at increasing CuSO_4_ concentrations (ratios of Cu (II):protein 0.5:1–2:1).

**Figure 4:**
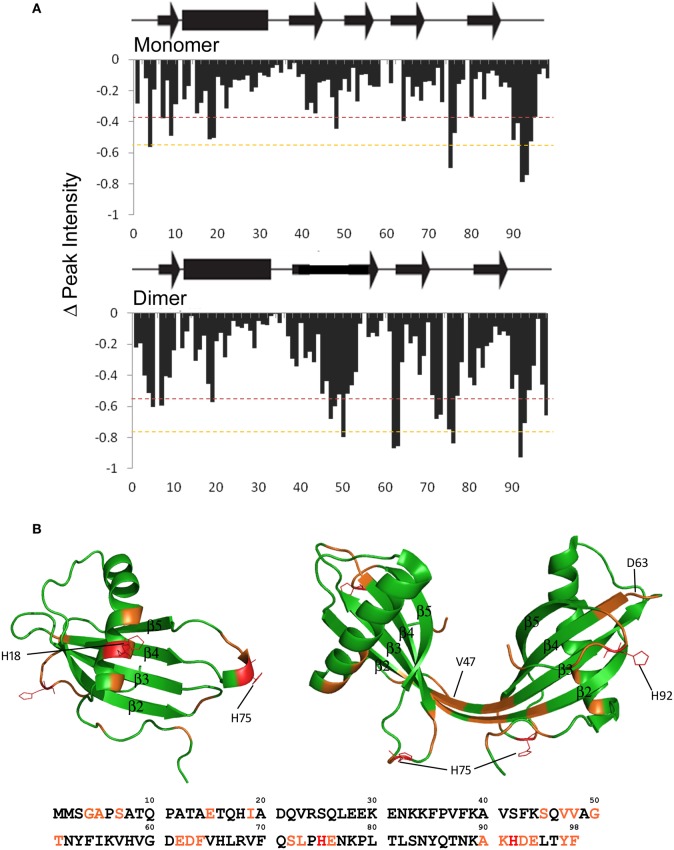
**Mapping copper binding sites in soluble stefin B. (A)** Changes in the relative intensity of amide NMR signals upon Cu^2+^ binding (calculated from the 2D ^1^H^15^N HSQC spectra). The dashed lines represent one (red) and two (orange) standard deviations from average intensity changes measured in solution. Changes above standard deviation are deemed to be significant. **(B)** Residues with significant changes are plotted in orange on the structures of the stefin B monomer and dimer. Histidine residues that are within these regions are drawn in red and are shown in stick form to highlight their position.

Figures 4A and B shows that there are three main regions in the monomeric state of stefin B that display a large reduction in peak height relative to the average change. These regions when modeled on the monomeric stefin B structure show two possible Cu (II) binding regions. Residues 15–22 which are within the α-helix facing away from the β-sheets form a likely binding area together with residues around the loop region of 75–77 (between β-strands 4 and 5). There are many residues within these two areas that are capable of binding a metal such as Cu (II), these being Glu15, His18, and Gln22 in the helix and His75, Glu76, and Asn77 in the loop region between β-strands 4 and 5. At first glance these regions seem too far apart to form a singular binding domain but the flexibility in the loop region where His75 is located may allow sufficient movement to bring the two histidine residues (18 and 75) together, and along with the other possible ligands in that region could form a site for Cu (II) coordination. The second possible Cu (II) binding region combines the residues at the C-terminal (90–94) with residues in the loop between β-strands 3 and 4 (residues 62–64). This site is not well defined in the monomeric ^1^H^15^N-HSQC data due to the lack of an assignment for residues 62 and 63, but when this binding site is looked at in the dimeric structure, where the assignment is available for this region, the data support these residues as belonging to this second binding site.

The equivalent peak height data for the dimeric state is shown in Figure 4A. The dimeric state shows a greater number of residues with reduced peak intensity compared to the monomer, though many of these are in the same area as in the monomer. The two loop regions containing residues 62–64 and 90–94 show a significant height loss in the spectra obtained on the addition of Cu (II). As mentioned previously there are multiple residues capable of binding Cu (II) in this region, again confirming the likelihood of these two regions forming a binding site. This is particularly obvious when viewed on the dimeric structure (Figure 4B). Residues 72–76 also show significant height reduction, similar to the monomer, but in the dimeric structure it appears that it no longer forms a binding site with the α-helix preferentially. The dimer shows an extra region (residues 45–51) that in the monomer is in the loop between β-strands 2 and 3 but in the dimer form the elongated β-strand that joins the two domains of the domain-swapped dimer. This region shows a greater number of residues displaying significant peak height change in the dimer than the α-helix does and so appears to replace the α-helix in forming a Cu (II) binding site with residues 72–76. There are also a few residues in the N-terminus that may contribute to this binding site, though they may just be in the vicinity of the bound Cu (II) ion. It is unclear why the elongated β-strand region (residues 45–51) would be a strong binding site for Cu (II) because there is a distinct lack of residues within that region capable of binding a metal ion (only Ser45 and Thr51). It is also worth noting that there is a slight alteration of the residues in the loop between β-strands 4 and 5 participating in Cu (II) binding shifting in the dimer when compared to the monomer. There is a shift toward residues close to the elongated β-strand (residues 72 and 73 in the dimer compared to residue 77 in the monomer).

The two possible binding sites that have been proposed here appear to have similar affinities for Cu (II). This can be deduced from the peak reductions during titration. If binding sites of unequal affinities were present the first site would bind up to a 1:1 stoichiometry [stefin B:Cu(II)] the second binding site would then be populated and become visible. In this data both binding sites appear simultaneously and so both must have similar affinities. This agrees with the previously published data (Zerovnik et al., 2006) which proposes two binding sites with picomolar affinities for Cu (II) (K_a_ = 7.2 × 10^10^ M^−1^ and K_a_ = 1.0 × 10^10^ M^−1^).

The specific regions of binding are thus defined and most likely incorporate residues other than the histidines proposed in previous work. We show here that His18 (monomer only), His75 and His92 are involved in Cu (II) binding but that His58 and His66 are not. We also show that there is a different Cu (II) binding mechanism employed by different oligomeric states of the protein (monomer and dimer). Unfortunately this data does not allow us to map specific interactions of Cu (II) binding because of the number of flexible loop regions involved. This means that exact residue side chain positions cannot be identified, only the region in which the Cu (II) has bound.

### Cu (II) retards the elongation of stefin B fibrils

Metal ions have different effects on different amyloid-forming proteins. Some studies find that metal ions speed up the rate of fibrillization or reduce the lag phase while others report that it retards the fibrillization process and in some cases can stop the process occurring completely (Atwood et al., 1998; Zou et al., 2001; Raman et al., 2005). The fibrillization time course of stefin B in the presence of Cu (II) was monitored using thioflavine T fluorescence. In order to interpret our results when comparing with the reaction in the absence of Cu (II), we first measured the effect of Cu (II) on the monomer and dimer structures of the protein under *in vitro* fibrillization conditions using 2D NMR spectroscopy (^1^H^15^N-HSQC). Although lowering the pH must affect the protonation of a number of residues identified as involved in coordinating Cu (II), the NMR peak intensities of stefin B are affected by Cu (II) in exactly the same way as seen under the more native pH conditions discussed in the previous section (data not shown). This confirms that both Cu (II) binding sites are retained at pH 4.7, most likely because protonation of coordinating residues is not yet significant.

**Figure 5:**
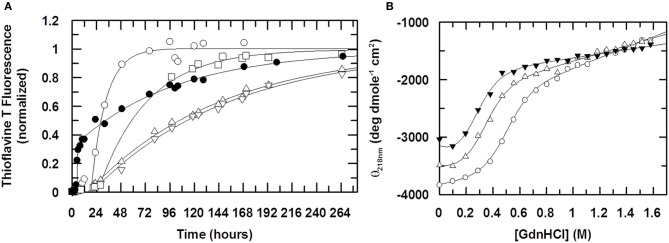
**The effect of Cu (II) on fibril formation.** In **(A)** the formation of stefin B fibrils is monitored using Thioflavine T fluorescence at 482nm (λ_*excitation*_ = 442 nm). The ratio of Cu (II) to protein is 0:1 (open circles), 0.5:1 (open squares), 1:1 (open triangles, up) and 2:1 (open triangles, down). The fibrillization of the mutant stefin B V59Q, where the equivalent point mutation to that seen in the amyloid disease-causing homolog, cystatin C (L68Q) is shown for comparison (closed circles). Solid lines represent data fitting to a single exponential increase. Elongation rate constants k_e_ are 1.9 × 10^−5^ s^−1^ in the absence of Cu (II) and 2.5 × 10^−6^ s^−1^ in the presence of saturating amounts. For the mutant V59Q (closed circles), the lag time is reduced 5-fold from 20 hours to 4 hours, whereas the elongation rate constant k_e_ is increased to 8 × 10^−5^ s^−1^. In **(B)**, the effect of Cu (II) on the stability of the folded state of stefin B under fibrillization conditions (15mM sodium acetate, 150mM NaCl, 10% TFE, pH4.7). The ratio of Cu(II):stefin B protein is 0:1 (open circles), 1:1 (open triangles, up), 5:1 (closed triangles). The calculated stabilities of the protein expressed as the free energy difference ΔG_F/U_ is −3.3, −2.2 and −1.7 kcal mole^−1^ respectively. Cu (II) binds preferentially and stoichiometrically to an unfolded form of stefin B, such that the observed slowing down of the fibrillization reaction in the presence of Cu (II) cannot come about through the stabilization of the folded state.

The fibrillization time course of stefin B shown in Figure 5A shows that Cu (II) retards the reaction by slowing the elongation phase of the reaction very significantly. The elongation rate constant k_e_ reduces by a factor of 10 from 1.9 × 10^−5^ s^−1^ in the absence of Cu (II) to 2.1 × 10^−6^ s^−1^ at a stoichiometric concentration of Cu (II). As Cu (II) is increased to a 2:1 (Cu (II):protein) stoichiometry, k_e_ does not change. The lag phase shows a slight change as Cu (II) is added but at its maximum are only 2-fold different to WT and so not a large enough difference to be significant.

Since above a ratio of 1:1 (Cu (II):protein) there is little or no increase in the effect on the rate of fibrillization, this suggests that the binding of Cu (II) during the fibrillization process is in a 1:1 stoichiometry. In the native form, at both pH 6.0 and 4.7, it has already been shown in this study that Cu (II) has two binding sites. If Cu (II) was altering the fibrillization kinetics through an interaction with the native form it would be assumed that Cu (II) would have maximal effect on the fibrillization process at a 1:2 stoichiometry. Therefore the effect of Cu (II) on the k_e_ of the fibrillization process may not be due to its interaction with the native form but instead its interaction with some other, partially folded form that has only a single Cu (II) binding site.

### Cu (II) favors an unfolded state of stefin B

To investigate the possibility that Cu (II) is interacting with a form of stefin B other than the natively folded one, the stability of stefin B with increasing concentrations of Cu (II) was investigated. The denaturing agent Guanidine hydrochloride (GdnHCl) was titrated into solutions of stefin B in the presence of increasing amounts of Cu (II) and circular dichroism used to detect changes in the fold of stefin B. The retardation of the fibrillization reaction may be due to the binding to and stabilizing of the native form by Cu (II) and so increasing the energy required to unfold to the appropriate degree to allow initiation of fibrillization. Alternatively, Cu (II) may be binding to another form of the stefin B and by binding is in turn stabilizing this form. There is a good chance, due to the fibrillization conditions being so close to the unfolding point of stefin B, that this unknown state is partially unfolded. Figure 5B shows the denaturation curve of WT stefin B along with the fitted parameters. At pH 4.7, stefin B is only marginally stable and at this pH has a very low midpoint of unfolding at 0.49 M GdnHCl. When Cu (II) is added we see that the unfolding curve is moved left, to a midpoint of unfolding of 0.32 M and 0.24 M GdnHCl denaturant activity for 50 μM and 250 μM Cu (II) (ratios of 1:1 and 5:1 Cu(II):protein) respectively. This suggests that Cu (II) is actually destabilizing the native structure at pH 4.7 rather than stabilizing it. The reduction in the midpoint of unfolding values during the titration (Figure 5B) suggests that Cu (II) is interacting with a partially folded or an unfolded form of stefin B under fibrillization conditions and therefore that it is not the stabilization of the natively folded state that is retarding the fibrillization process. Electron microscopy data confirm that the fibrils produced in the absence and presence of Cu (II) have similar morphology (data not shown).

## Discussion

So what changes could be responsible for producing different precursor states? The work presented here highlights particular regions of the stefin B protein that are affected at the local level when conditions that either favor or hinder amyloid fibril formation are titrated in. Under all the conditions monitored here, the overall structure of the stefin B retains its native fold and therefore the structured areas are less sensitive to the titrations than the more intrinsically flexible loops. We will consider here how changes in these loops may trigger secondary structure re-arrangements and eventually lead to new tertiary folds.

### The role of the helix (residues 13–33)

Previous work (Janowski et al., 2001; Staniforth et al., 2001; Morgan et al., 2008) on stefin B and other cystatins has shown that the displacement of the helix from the β-sheet is key to the formation of dimers and amyloid fibrils. In fact the hereditary amyloidosis associated with cystatin C (Palsdottir et al., 1988; Levy et al., 1989) is caused by a point mutation at position 68 which is thought to weaken the interaction between the helix and the β-sheet (Janowski et al., 2001). This position is aligned with valine 59 in the stefin B protein studied here (Rawlings and Barrett, 1990). Mutating this residue to glutamine in the same way causes a V59Q protein with accelerated fibrillization kinetics compared to that of the WT (Figure 5A) while it is otherwise structurally very similar. Although *in vivo* effectors of fibrillization appear to directly affect the helix, can the fibrillization conditions used *in vitro* be having a similar effect?

Cu (II) is observed to bind to two regions of the protein and one of these sites changes when the protein dimerizes (Figure 4). In the monomeric form of the protein, Cu (II) binds between the helix and the loop between strands 4 and 5 (residues 75–77). As the protein dimerizes, the helix alters this interaction and its significance in the binding of Cu (II) is lessened. The loop between 4 and 5 is now involved in forming a site with residues in the loop between strands 2 and 3 (residues 45–51) which becomes part of an elongated β-strand. This change in focus for metal-binding reflects on the helix, a key area important for both dimerization and fibrillization reactions.

The mechanism by which modifications in this area may specifically lead to fibrillization must be a combination of events but most probably starts with an increased flexibility in the loops either end of the helix. It is notable in this optic, that both the N-terminal and the loop between the helix and strand 2 contain proline at positions 6, 11, and 36. Just as the other two prolines in the protein, these residues are in the trans conformation in the crystal structure of the monomer, but may isomerize in solution, especially during the partial unfolding involved in dimerization. Proline 6 is in the unstructured N-terminal region (Figure 2B), and nearby residues show two peaks in the NMR spectrum of the monomer. This may be responsible for the multiple peaks for the N-terminal residues in the dimer (Figure 2C), although the effect is more pronounced in this form of stefin B, suggesting that another process may be involved. It is possible that isomerization of proline 11 is also playing a role. Proline 36 is in a loop region between the α-helix and the second strand of the β-sheet. Several nearby regions have multiple peaks, and the packing of these residues is shown in Figure 2D. Most of these residues have two peaks in the ^1^H^15^N-HSQC spectrum, so proline isomerization could account for both of these. In support of a role for these regions is the observation that a mutant stefin B (G4R) associated with the degenerative form of epilepsy EPM-1 has a 4-fold longer lag phase (Rabzelj et al., 2005). Also, a possible polymorph of stefin B where glutamate at position 31 is replaced by a tyrosine retards fibrillization while destabilizing the native state substantially (Kenig et al., 2006).

### The C-terminal (residues 90–98) and the loop between strands 3 and 4 (residues 60–62)

The family I cystatins or stefins contain a very short sequence (three residues) between strands 3 and 4 unlike the family II cystatins such as cystatin C, where an extended, disulphide-bridged loop is present (22 residues). The work presented here highlights this region in the protein as affected by the mildly acidic conditions necessary for fibrillization (Figure 3A) but also a key to Cu (II) binding in monomers and dimers within a site possibly involving nearby residues in the C-terminal (Figure 4). Again this latter part of the protein is different in the stefins, where compared with the single residue observed in the family II cystatins, this area of the protein is an extended conformation with restricted chain flight (nine residues), where the backbone of the terminal residue 98 loops back to hydrogen bond with the side chain amide of residue 84 in strand 5 (Stubbs et al., 1990; Jenko Kokalj et al., 2007; Morgan et al., 2008). However in all cystatins, albeit in different ways, these regions are close enough to affect each other, and together with the loop between strands 4 and 5 may be important in modulating the structural outcome of strands 4 and 5.

### The domain-swapping “hinge” region (residues 45–51)

3D domain-swapping in the cystatin proteins involves the formation of dimers where the cystatin fold is retained but is made of two different polypeptide chains. Residues 1–47 are contributed by one chain while residues 48–98 from a separate chain make up the remainder of the fold (Janowski et al., 2001; Staniforth et al., 2001; Jenko Kokalj et al., 2007). The “hinge” bridging the 2 cystatin folds is the former active site loop of the protease inhibitor (residues 45–51) which now relaxes into an extended β-sheet structure. Strands 2 and 3 of the original fold now form an unusually long, continuous β-strand which is 20 residues long (residues 37–57). This feature of the dimers appears to be retained in the amyloid fibrils made from stefin B (Morgan et al., 2008) where protection from hydrogen-exchange and therefore secondary structure can be mapped to the whole length of this strand. This part of the molecule also contains the regions with the highest degree of amyloidogenicity according to predictor programs (for e.g., Conchillo-Sole et al., 2007; Tartaglia et al., 2008; Maurer-Stroh et al., 2010), with residues central to strand 3 (and to a lesser extent to strand 5) are identified as likely to be core to the new amyloid structure.

It is intriguing therefore that the hinge region core to this structure is strongly affected by fibrillization conditions. As explained in the previous section, Cu (II) favors binding to the dimeric conformation of this hinge (Figure 4C) whereas the amyloidogenic solvent TFE destabilizes it (Figure 3B). This suggests that although this region may be retained in a dimer-like extended β-strand conformation in the amyloid fibrils, flexibility in this region is required to enable successful assembly of amyloid. This would be the case for example if propagated 3D domain-swapping were the key to assembly as proposed for human cystatin C (Wahlbom et al., 2007) but also supports any model requiring significant further tertiary re-arrangements (Morgan et al., 2008).

Although discounted as the least likely proposition, it is formally possible that the hinge region in stefin B may be responsible for the multiple discrete peaks observed in the dimer NMR spectrum (^1^H^15^N HSQC) of stefin B (Figure 2). Constraints on the flexibility of the hinge, unlike in its stefin A counterpart where there appears to be none, may be restricting the conformational space available to the protein as it assembles into larger oligomers.

### The role of the loop between strands 4 and 5 (residues 71–79)

The residues that have been identified to stabilize the tetrameric form have also been identified as the residues showing chemical shift change in fibrillization conditions (Figures 3 and 4). In fact, the whole loop involved in the “hand shaking” tetramerization interface containing residues P74 and H75 (Jenko Kokalj et al., 2007) is likely to be altered under fibrillization conditions since residues in the loop 72–79 display chemical shift change. In order to interpret the effect of these changes, it is important to consider the role of the prolines at positions 74 and 79. A trans- to cis- isomerization of the backbone amide bond at the conserved proline 74 position is thought to be key to the formation of the new inter-dimer interface created in the tetramer.

The mutation of two proline residues (74 and 79) to serine creates proteins which both display altered characteristics (Jenko Kokalj et al., 2007; Smajlović et al., 2009). One allows the stabilization of tetrameric form (P79S) while the other (P74S) drastically slows the kinetics of fibrillization. These results taken together implicate the conformation of the loop containing residues 72–79 as having a major influence in the oligomerization of stefin B.

Cu (II) is shown to retard elongation of fibrillization of stefin B and is shown to interact with the loop containing P74 and P79 in both monomeric and dimeric stefin B. Data presented in Figure 5 suggest that a further modification of this site, although slowed by the presence of Cu (II) may result in a new interaction with Cu (II) which stabilizes unfolded forms of stefin B (Figure 5B) and eventually fibrillar forms (Figure 5A). At the same time, protonation of Histidine 75 is key to changes observed in the protein as the pH is lowered and favors fibrillization reactions.

Prolines 74 and 79 are on either side of the second active site loop and are likely to be the source of the multiple peaks observed in strand 5 of the dimer (Figures 2B and C). They could represent the alternate conformations of the β-strands and the intervening loop when one, both or neither proline is in the *cis* conformation. The single peaks observed for residues in the loop itself could be due to the flexibility of the loop causing conformational averaging in this region. As shown in Figure 2E, perturbations in strand 5 would also affect the conformation of phenylalanine 98, whose side chain packs against the aliphatic part of the side chains of asparagine 84 and aspartate 86.

### The role of different structural states in the dimer of stefin B

It is possible that all the multiple peaks observed may be due to alternative packing arrangements of the both N-terminal region and the loop between strand 4 and 5, which in turn are governed by the conformation of the proline residues in these regions. The single peaks observed in the spectrum of the tetramer suggests that this species populates a single form (or that conformational averaging results in single peaks) possibly selected from one of the dimer conformations.

The regions of the stefin B dimer which have multiple peaks in its ^1^H^15^N HSQC spectrum (Figures 2B and C) are similar to those that show a conformational change between the monomer and tetrameric crystal structures. The two active site loops of the monomer appear to be able to access several conformations, and it is these regions which are important in the tetramerization reaction. It is possible that a particular conformation of these regions is necessary for dimers to associate into tetramers, and that only a subset of dimer conformations (perhaps one of the four states observed in solution) can form tetramers. Since the multiple states in the dimer appear to be due to proline isomerization, which is also responsible for the orientation of the loop between strands 4 and 5, then this process may well control the dimer-tetramer equilibrium.

It is possible that similar arguments may be valid regarding the ability of the dimer to assemble into amyloid fibrils if the tetramer represents a “dead-end” in a reaction that can otherwise propagate. This view is supported by data on chicken cystatin, where indistinguishable unimolecular processes (rearrangement within the dimer) are rate-limiting for the dimer to tetramer and dimer to amyloid fibril reactions (Sanders et al., 2004 and manuscript in preparation). However, without more detailed kinetic data on this process for stefin B, it is not possible to confirm this hypothesis.

The theory of uncoupled protein folding/unfolding being involved in the fibrillization is not just confined to stefin B. Other proteins that form amyloid fibrils do so under destabilizing but non-denaturing conditions, conditions that could easily destabilize some regions more than others, leaving particular interactions required for fibrillization intact. For example, the fibrillization of β_2_-microglobulin at pH 3.6 occurs above the midpoint of unfolding for this protein (McParland et al., 2000). However, looking specifically at this protein, the incorporation of Cu (II) induces fibrillization at physiological pH (Eakin and Miranker, 2005). This has been shown to be due to interactions of Cu (II) with a specific region inducing the formation of a native like structure that retains its high β-sheet content but has alternative conformations in localized loop regions (Eakin et al., 2006). This region is also a loop containing a proline residue which has striking parallels with the observations reported here. This all confirms the importance of localized regions and shows that conditions that induce fibrillization merely help the protein adopt the required conformational change more frequently.

## Conclusion

Viewing these results as a whole suggests that the zigzag interaction of loop regions (particularly the loop 72–79) may be responsible for bringing multiple stefin B protein chains together. During this time uncoupled unfolding, particularly the detachment of the α-helix, may occur. This partially unfolded but possibly still domain-swapped dimeric core structure may represent the nucleus of the fibrillization process. This would allow the interaction of the β-sheet region of multiple stefin B chains resulting in the initiation of amyloid formation or in the attachment to an existing fibril. The interaction of the β-sheet of multiple protein chains would leave the helix excluded and exposed to the solvent which is consistent with the proposed structural model of stefin B fibrils (Morgan et al., 2008). However, not all loop conformations would adapt easily to the growing fibril. A selection would be made from the conformations divided by relatively high energetic barriers of proline isomerization between cis or trans. For example, in the tetramer only the cis isomer of P74 is allowed (Jenko Kokalj et al., 2007). Similarly, some specific isomeric state could be fixed in the fibrils. This further underlines the proposed role for peptidyl prolyl isomerases (PPIs) in amyloid diseases (Eakin et al., 2006; Jahn et al., 2006; Pastorino et al., 2006; Ryo et al., 2006). This suggests prolines act as switches between off-pathway oligomers and on-pathway fibrillar states.

### Conflict of interest statement

The authors declare that the research was conducted in the absence of any commercial or financial relationships that could be construed as a potential conflict of interest.
